# Prenatal Diagnosis of c.437–1G>A Mutation in the MAN2B1 Gene in a Family With Alpha-Mannosidosis: Unraveling Clinical Presentation and Treatment Outcomes in a Novel Prenatal Case

**DOI:** 10.7759/cureus.58922

**Published:** 2024-04-24

**Authors:** Talal AlAnzi, Sarar Mohamed, Amal AlHashem, Hadeel AlRukban

**Affiliations:** 1 Pediatrics, Johns Hopkins Aramco Healthcare, Dhahran, SAU; 2 Division of Genetics and Metabolic Medicine, Department of Pediatrics, Prince Sultan Military Medical City, Riyadh, SAU

**Keywords:** case report, prenatal diagnosis, man2b1 gene, lysosomal storage diseases, alpha-mannosidosis

## Abstract

Alpha-mannosidosis is a rare lysosomal storage disorder with progressive impairments in motor functions, skeletal deformities, and immunodeficiency. Enzyme replacement therapy (ERT) should be initiated early to achieve optimal outcomes. This report describes how alpha-mannosidosis diagnosis in a seven-year-old girl led to a successful prenatal diagnosis in the subsequent pregnancy and pre-symptomatic treatment at the early disease stage. The index patient was a seven-year-old girl who was referred with a confirmed diagnosis of alpha-mannosidosis based on the presence of homozygous c.437-1G>A mutation in the MAN2B1 gene. A prenatal diagnosis was made in the subsequent pregnancy through molecular analysis, which revealed the same homozygous variant. The patient was treated at the fifth week of age and showed mild skeletal involvement and normal development at ERT initiation. At 11 months of age, the ERT level increased to 15.8 µmol/l/h. The motor assessment showed that the patient was developmentally normal and was able to maintain her sitting and walking for a few steps only. Prenatal molecular screening in affected families can allow for the early identification and implementation of appropriate management strategies for alpha-mannosidosis.

## Introduction

The lysosomal alpha-d-mannosidase (EC 3.2.1.24), encoded by the MAN2B1 gene in two major forms, is an essential enzyme in the degradation pathway of N-linked glycoproteins within lysosomes, playing a critical role in the catabolism of glycoproteins and glycolipids. This enzyme catalyzes the hydrolysis of terminal alpha-linked mannose residues under an acidic pH optimum to prevent the accumulation of oligosaccharides in several tissues [1]. Thus, deficient lysosomal alpha-d-mannosidase activity can result in a lysosomal storage disorder (LSD), alpha-mannosidosis, characterized by progressive impairments in motor and neurological functions, skeletal deformities, and immunodeficiency [2,3]. Alpha-mannosidosis (OMIM 248500) is a rare autosomal recessive LSD with a birth incidence ranging from 1:600,000 to 1:1,042,000 [1]. The condition is caused by mutations in the MAN2B1 gene. To date, 154 variants have been identified in the MAN2B1 gene, including missense mutations, nonsense mutations, deletions, insertions, and splice site mutations [4-6]. Missense and nonsense mutations are the most commonly reported variants in alpha-mannosidosis patients, resulting in unstable or truncated and nonfunctional alpha-d-mannosidase proteins and several clinical phenotypes [4].

Alpha-mannosidosis is a progressive disorder with a wide range of clinical features and is typically classified into three types according to the age of onset and severity. Patients with type I, a mild phenotype, often present in the second or third decade of life and show a slower disease progression than the other types [1,7]. On the other hand, type II alpha-mannosidosis is characterized by intermediate disease severity and typically present in early childhood with developmental delay, intellectual disability, hearing impairment, speech difficulties, skeletal abnormalities, and recurrent infections [8]. Type III alpha-mannosidosis is a rapidly progressing phenotype that presents in infancy or early childhood, with severe skeletal and neurological abnormalities, such as ataxia, nystagmus, and seizures. Patients with type III disease usually suffer from an early death due to neurological involvement or myopathy [1,7]. The diagnosis of alpha-mannosidosis requires the presence of reduced alpha-d-mannosidase enzyme activity (5-15% of normal activity) in leukocytes or other nucleated cells using fluorometric or spectrophotometric assays [8]. Molecular sequencing can then confirm the diagnosis by identifying the disease-causing variants in the MAN2B1 gene [9].

The treatment options for alpha-mannosidosis are currently limited, and management primarily focuses on supportive and symptomatic care. In selected cases, hematopoietic stem cell transplantation (HSCT) offers benefits in preventing or slowing the progression of neurocognitive function and early death [10]. However, HSCT carries significant risks, including graft-versus-host disease, pulmonary complications, and transplant-related mortality [10-12]. In 2018, enzyme replacement therapy (ERT) was approved to treat non-neurological manifestations of moderate alpha-mannosidosis [1]. Results from phase I-III trials showed that ERT significantly improved biochemical markers, motor function, and pulmonary functions in patients with alpha-mannosidosis [13-15]. These trials showed greater benefits in pediatric age groups, supporting the benefits of ERT initiation at the early stage of the disease [16].

In this case report, we describe how the diagnosis of alpha-mannosidosis in a 7-year-old girl led to successful genetic counseling, prenatal diagnosis of homozygous c.437-1G>A mutation in the MAN2B1 gene at the subsequent pregnancy, and pre-symptomatic treatment at early disease stage.

## Case presentation

The present manuscript was prepared according to the CARE (CAse REport) guidelines [17].

Case I: the index patient

A seven-year-old girl was referred to our center in January 2021 with a diagnosis of alpha-mannosidosis based on the presence of homozygous c.437-1G>A mutation in the MAN2B1 gene. The patient had a history of speech delay, intellectual disability, recurrent otitis media following tympanostomy, and scoliosis since the age of five. Her records also showed a history of right hip surgery for developmental dislocation, multiple adenoidectomies, and surgical repair of umbilical and inguinal hernias. The family history revealed that the parents were first-degree cousins, and the patient had a healthy four-year-old sister. There was a history of intellectual disability in the extended family.

**Figure 1:**
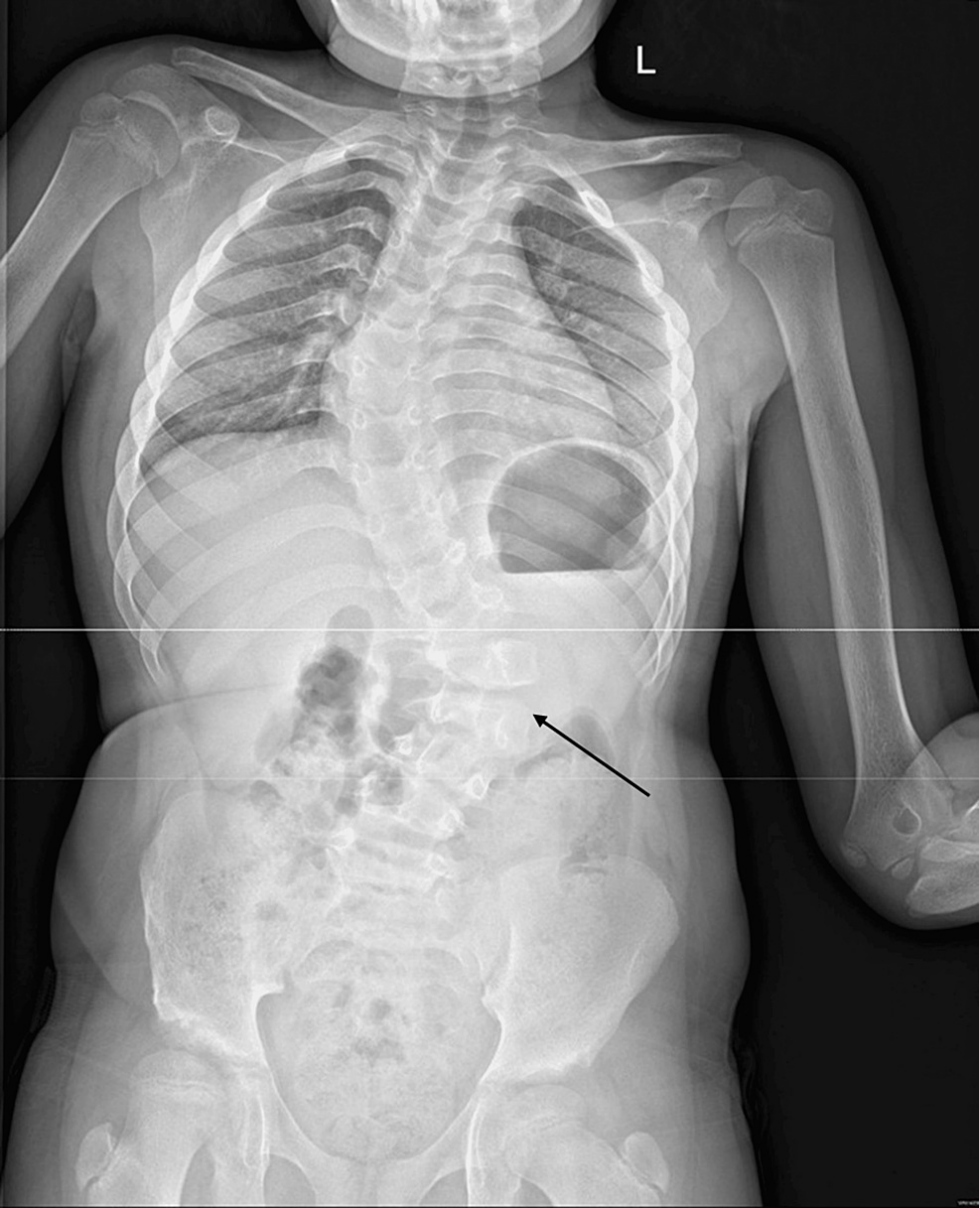
X-ray of the whole spine shows re-demonstration of S-shaped scoliosis. The dorsal dextroscoliosis Cobb's angle measures approximately 47 degrees; the lumbar levoscoliosis Cobb's angle measures approximately 35.7 degrees.

Upon physical examination, the patient exhibited dysmorphic features characterized by coarse facial characteristics such as frontal bossing, supra-orbital bridge prominence, thick eyebrows, a bulbous nose, and thick lips. Her head circumference was 51 cm (70-90th percentile), and her weight was 27 kg (90th percentile). While the vital signs were unremarkable, the skeletal survey displayed evidence of dysostosis multiplex, multilevel lumbar fusion, vertebral anomalies, and S-shaped scoliotic thoracolumbar deformity (Figure 1).

Neurological examination revealed an ataxic gait, intact cranial nerves, and normal muscle tone and reflexes. Cardiac echocardiography indicated a trivial mitral valve prolapse, mild regurgitation of the anterior leaflet, and mild left ventricular hypertrophy. Furthermore, the abdominal ultrasound demonstrated hepatosplenomegaly. Brain magnetic resonance imaging (MRI) showed no abnormalities. Polysomnography results showed no signs of obstructive sleep apnea, and the ENT examination was unremarkable. The enzymatic fluorimetry assay showed reduced alpha-d-mannosidase activity (8.6 µmol/l/h, normal range ≥ 16,2 µmol/l/h).

**Figure 2:**
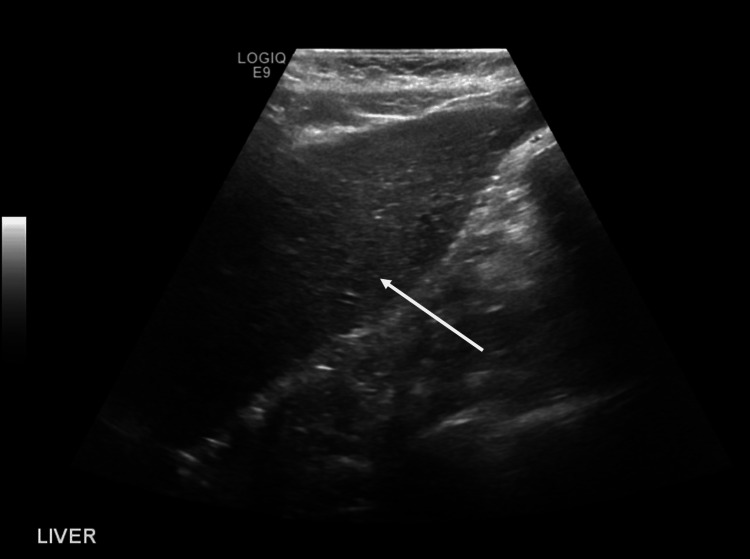
Ultrasound image of the patient's liver. The liver is normal in size measures 9.9 cm, with normal homogeneous parenchymal echogenicity and smooth surface outline. No obvious focal hepatic lesion could be seen.

Therefore, the patient initiated ERT and demonstrated a good response one year after treatment. The motor assessment after one year showed adequate muscle strength (3/5), and the patient was capable of walking independently, albeit with a limp, climbing stairs with some difficulty, and running for brief distances. She was nearly independent in performing daily activities. She demonstrated the ability to write her name and draw lines and shapes. The 30-meter walkway length for the six-minute walk test (6MWT) revealed that the patient was able to walk for 60 steps while holding the rail and the father without an elevated heart rate. In terms of speech, the patient had a limited vocabulary of fewer than 20 words and was unable to form sentences. The intelligence quotient (IQ) assessment was 80, necessitating a special needs school. The enzymatic fluorimetry assay showed normal alpha-d-mannosidase activity (37.4 µmol/l/h), and the urine analysis using chromatography showed a slightly abnormal increase in the 2-hydroxyisovaleric acid. The abdominal ultrasound showed normal sizes of the liver and spleen (Figure 2).

Case 2: the prenatally-diagnosed patient

Based on the confirmed diagnosis of the index patient, the genomic DNA of the fetus of the subsequent pregnancy was obtained from chorionic villi and cultured amniocytes. The mutational analysis was performed using the polymerase chain reaction-restriction fragment length polymorphism (PCR-RFLP) technique. The molecular analysis revealed the presence of the homozygous NM_000528.3:c.437-1G>A variant in the MAN2B1 gene. The molecular analysis was repeated at the fifth week of age and revealed the same mutation.

**Figure 3:**
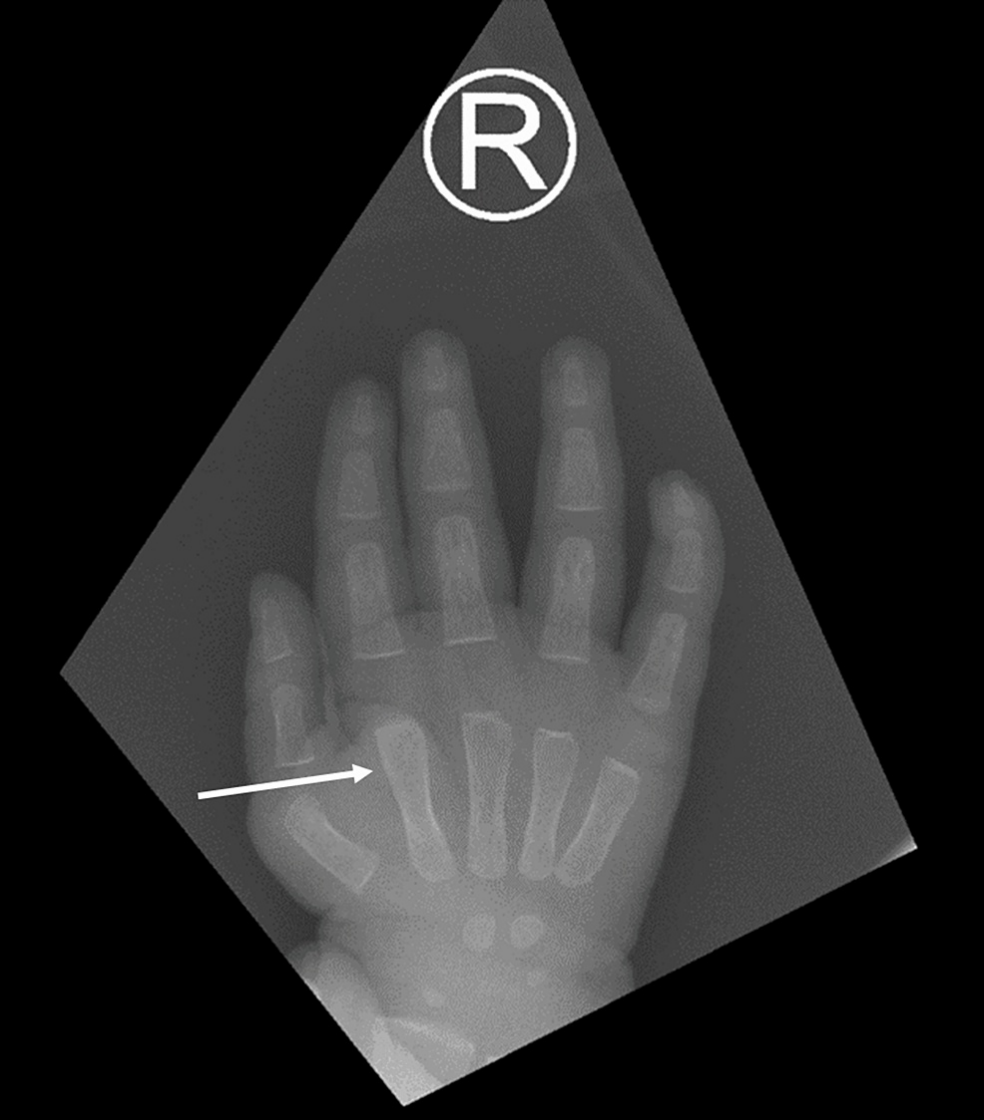
An X-ray of the right hand showing a mild widening of the short bones.

At the fifth week of age, the patient showed unremarkable findings on physical examination, normal developmental milestones, and normal range of motion. The skeletal survey showed absent pneumatization and hypoplastic appearance of the maxillary antra, reduced pneumatization of the mastoid air cells, and hypoplastic frontal sinus. Additionally, there were thickened frontal and occipital bones of the skull. The odontoid process was hypoplastic, with a tendency for atlantoaxial subluxation, and there was potential anterior breaking of lumbar vertebral bodies. A mild widening of the short bones in the hand was noted (Figure 3).

Hypoplastic inferior aspects of the iliac bones led to acetabular dysplasia and the possibility of developmental dysplasia of the hip (DDH). The abdominal ultrasound showed mild hepatomegaly, and the echocardiography showed mild tricuspid valve insufficiency. On the other hand, the brain MRI and sleep report were normal. The enzymatic fluorimetry assay showed reduced alpha-d-mannosidase activity (<6.2 µmol/l/h). The patient started ERT at the age of five months. At 11 months of age, the enzyme, alfa mannosidase, level increased to 15.8 µmol/l/h. The motor assessment showed that the patient was developmentally normal and was able to maintain her sitting and walking for a few steps only. The serum IgG level was normal after one year of ERT.

## Discussion

To our knowledge, this is the first case report that describes successful genetic counseling and prenatal molecular diagnosis of homozygous c.437-1G>A mutation in the MAN2B1 gene in a fetus with a family with alpha-mannosidosis. We demonstrated that prenatal PCR sequencing was feasible and resulted in an accurate diagnosis of alpha-mannosidosis. Early ERT initiation was associated with significant improvements in alpha-d-mannosidase activity, normal motor development and cognitive function, and prevention of progressive skeletal deformities. Such findings highlight the crucial role of prenatal molecular testing in families with a history of homozygous mutations in the MAN2B1 gene. The present report also shows the benefits of ERT in pediatric cases with moderate alpha-mannosidosis. The initiation of ERT in the seven-year-old girl was associated with significant improvements in motor and cognitive functions, as well as prevention of further progression in skeletal deformities.

The introduction of ERT has significantly improved the management landscape of several LSDs, including mucopolysaccharidoses (MPS), Gaucher disease, and alpha-mannosidosis [18]. Early treatment initiation is crucial for the success of ERT in LSDs. Several studies have demonstrated that early initiation of ERT leads to better clinical outcomes and can prevent or delay the onset of irreversible tissue damage and organ dysfunction [19]. In the setting of alpha-mannosidosis, it was found that ERT had greater benefits in pediatric age groups, supporting the benefits of ERT initiation at the early stage of the disease [16]. Therefore, prenatal molecular diagnosis has emerged as a valuable tool for the early detection of LSDs, including alpha-mannosidosis, in families with a known history of these disorders. Identifying pathogenic mutations in the fetus once the index case is confirmed can provide valuable information on the risk of developing LSDs, enabling families to make informed decisions about early interventions [20]. With the advances in sequencing technologies, prenatal screening of known and novel mutations has become more accessible for routine clinical applications [21]. In this report, we demonstrated the feasibility of prenatal identification of a pathogenic variant in the MAN2B1 gene in a family with an index case of alpha-mannosidosis. A previous report by Verma et al. assessed the feasibility of molecular diagnosis in 43 fetuses with biochemically diagnosed LSDs. The prenatal molecular screening showed high accuracy with one false positive case [21]. A more recent report showed that combining enzymatic and molecular testing improved the accuracy of prenatal screening of LSD [22].

The present report demonstrated the effectiveness and safety of ERT for both infantile and pediatric cases with alpha-mannosidosis in routine clinical practice. Both cases in the present report showed notable delay or prevention of the onset of skeletal deformities and organ dysfunction, as well as normal motor development in the infantile case and improved motor functions in the pediatric case. The efficacy of the recombinant human alpha-mannosidase, velmanase alfa, has been well-characterized in the rhLAMAN clinical program. The rhLAMAN phase I-II trial results showed that ERT significantly improved biochemical markers, motor function, and pulmonary functions over 12 months in patients with alpha-mannosidosis [13]. In the pivotal phase III rhLAMAN-05 trial, velmanase alfa significantly improved the 3-minute stair climb test (3MSCT) after 12 months of treatment. Additionally, the treatment group experienced improvements in other functional and biochemical endpoints, such as the 6-MWT, forced vital capacity, and serum oligosaccharide levels [14]. A subsequent open-label trial extension for up to four years showed sustained improvements in the 3MSCT, 6-MWT, and forced vital capacity. Moreover, the treatment was well-tolerated, with no serious adverse events related to the study drug reported [15]. Interestingly, a phase II trial of patients under the age of six showed that ERT led to significant improvements in motor function, joint mobility, and quality of life, as well as reductions in the serum and urine oligosaccharide levels following >24 months of treatment [23].

Several pathogenic variants have been identified in the MAN2B1 gene, including missense mutations, nonsense mutations, splice-site mutations, and deletions or insertions. Missense variants resulting in amino acid substitutions, such as c.2248C>T and c.458G>T, are the most common variants associated with alpha-mannosidosis [24]. However, the diverse nature of these variants and their consequences on the alpha-D-mannosidase enzyme contributes to the phenotypic heterogeneity observed in the disease [4,24]. On the other hand, splice-site variants of the MAN2B1 gene are less reported in alpha-mannosidosis and can lead to a nonfunctional or unstable enzyme. This report showed a novel splicing variant, c.437-1G>A, in the MAN2B1 gene. This mutation has not been reported before [25].

## Conclusions

The present case report highlights the importance of prenatal molecular screening in families with a history of alpha-mannosidosis, allowing for early identification of affected fetuses and implementation of appropriate management strategies. Prenatal diagnosis and early enzyme replacement therapy initiation allowed normal motor development, cognitive functions, and limited skeletal involvement. Further research is needed to assess the genotype-phenotype correlations to better predict the clinical course and treatment response in affected individuals.
